# Socioeconomic determinants and reasons for non-acceptance to vaccination recommendations during the 3^rd^ - 5^th^ waves of the COVID-19 pandemic in Hungary

**DOI:** 10.1186/s12889-024-19267-2

**Published:** 2024-07-05

**Authors:** Anna Sára Ligeti, Beatrix Oroszi, Csaba Luca, Edit Bilics, József Ágoston, Gergely Röst, Júlia Koltai

**Affiliations:** 1 National Laboratory for Health Security, HUN-REN Centre for Social Sciences, Budapest, Hungary; 2 National Laboratory for Health Security, Center for Epidemiology and Surveillance, Semmelweis University, Budapest, Hungary; 3 Corvinus University of Budapest, Budapest, Hungary; 4 National Laboratory for Health Security, Bolyai Institute, University of Szeged, Szeged, Hungary; 5 Faculty of Social Sciences, Eötvös Loránd University, Budapest, Hungary

**Keywords:** Vaccination hesitancy, Vaccine coverage, COVID-19, Cross-sectional survey, Socioeconomic determinants

## Abstract

**Background:**

In Hungary, although six types of vaccines were widely available, the percentage of people receiving the primary series of COVID-19 vaccination remained below the EU average. This paper investigates the reasons for Hungary’s lower vaccination coverage by exploring changing attitudes towards vaccination, socio-demographic determinants, and individual reasons for non-acceptance during the 3^rd^ - 5^th^ pandemic waves of COVID-19.

**Methods:**

The study’s empirical analysis is based on representative surveys conducted in Hungary between February 19, 2021, and June 30, 2022. The study used a total of 17 surveys, each with a sample size of at least 1000 respondents. Binomial logistic regression models were used to investigate which socio-demographic characteristics are most likely to influence vaccine hesitancy in Hungary. The study analysed 2506 open-ended responses to identify reasons for vaccine non-acceptance. The responses were categorised into four main categories and 13 sub-categories.

**Results:**

Between the third and fifth wave of the pandemic, attitudes towards COVID-19 vaccination have significantly changed. Although the proportion of vaccinated individuals has increased steadily, the percentage of individuals who reported not accepting the vaccine has remained almost unchanged. Socio-demographic characteristics were an important determinant of the observed vaccine hesitancy, although they remained relatively stable over time. Individuals in younger age groups and those with lower socioeconomic status were more likely to decline vaccination, while those living in the capital city were the least likely. A significant reason behind vaccine refusal can undoubtedly be identified as lack of trust (specifically distrust in science), facing an information barrier and the perception of low personal risk.

**Conclusion:**

Although compulsory childhood vaccination coverage is particularly high in Hungary, voluntary adult vaccines, such as the influenza and COVID-19 vaccines, are less well accepted. Vaccine acceptance is heavily affected by the social-demographic characteristics of people. Mistrust and hesitancy about COVID-19 vaccines, if not well managed, can easily affect people’s opinion and acceptance of other vaccines as well. Identifying and understanding the complexity of how vaccine hesitancy evolved during the pandemic can help to understand and halt the decline in both COVID-19 and general vaccine confidence by developing targeted public health programs to address these issues.

**Supplementary Information:**

The online version contains supplementary material available at 10.1186/s12889-024-19267-2.

## Introduction

Vaccination has been proven effective in mitigating the health and societal impacts of COVID-19 globally and preventing millions of fatalities [1]. However, in Hungary, the cumulative percentage of people receiving the primary series of COVID-19 vaccination was about 11% below the European Union average, and the percentage receiving at least one booster dose of vaccination was 18% below the EU average among the adult population in 2023 (Figure 4 of the Supplementary Material) [2].

In Hungary, both vaccination recommendations and six types of COVID-19 vaccines were widely available during the COVID-19 vaccination campaign in 2021-2022 [3], providing individuals with wide access, free of charge vaccines and a choice of vaccine types to increase vaccination uptake [4]. Although the vaccination campaign initially achieved success, with vaccination coverage exceeding the EU average until August 2021, vaccination coverage did not significantly increase since early 2022, when it reached 71% in the adult population (refer to Figure 4 in the Supplementary Material) [2]. As a result, a considerable segment of the Hungarian population has been left unvaccinated against COVID-19. The social patterning of COVID-19 vaccination coverage in Hungary since the third pandemic wave has hindered effective epidemic control. Primarily urban, less deprived areas have had the highest coverage, whilst the most deprived areas have had the lowest [3]. Despite recommendations for vaccination, widespread vaccine hesitancy could be the reason for non-vaccination.

Vaccine hesitancy refers to delayed uptake or refusal of vaccination despite the availability of vaccination services [5]. Underimmunisation and vaccine hesitancy is a major public health concern [6]. In 2019, the World Health Organisation (WHO) listed vaccine hesitancy as one of the top ten global health threats [7]. Although the prevalence of vaccine hesitancy was increasing before 2020 [8], the COVID-19 pandemic has exacerbated this issue. It has, probably resulted in a high number of deaths, many of which could have been saved if people had followed vaccination recommendations [1].

The Health Behaviour Model (HBM), the 3C, 5C and 7C models provide insight into the reasons behind vaccine hesitancy. The HBM’s investigation of correlations between health and preventive behaviours, 3Cs model developed by WHO’s Strategic Advisory Group of Experts on Immunization (SAGE) building on three factors with Complacency (perceived risks of the disease, vaccination as a non-priority), Convenience (availability, accessibility, affordability, health literacy) and Confidence (trust in vaccines, safety, delivery, and policy makers), moreover the further developed 5Cs model’s supplemet with Collective responsibility (social norms, willingness to protect others) and Calculation (seeking information before the decision) [9, 10]. The 7C model incorporates two additional factors, Compliance and Conspiracy [11].

Several European countries have reported a high level of COVID-19 vaccine hesitancy, due to demographic factors, poor health literacy, concerns about vaccine effectiveness and safety and mistrust of government and scientific institutions [12–16]. Data collected in the European Union shows that trust in science is negatively correlated, while trust in social media is positively associated with vaccine hesitancy [17]. A cohort study conducted in Hong Kong and Singapore between 2020 and 2022 found that four key factors were associated with vaccine refusal in both the 18-59 and over 60 age groups. These factors were mistrust in health authorities, low vaccine confidence, vaccine misconceptions, and political views [18]. A Canadian study highlighted the importance of trust in relation to vaccine hesitancy. It found that individuals with high levels of vaccine hesitancy also had significantly lower levels of institutional trust [19].

The reasons for non-acceptance of vaccination recommendations and their prevalence in the population may vary across countries [15]. Therefore, it is necessary to investigate the underlying reasons behind Hungary’s substantially lower COVID-19 vaccination coverage compared to the EU average.

The aim of this study is to describe the changing attitudes towards COVID-19 vaccination over time during the 3^rd^ - 5^th^ pandemic waves of the COVID-19, while investigating the socio-demographic determinants and the individual reasons for non-acceptance of COVID-19 vaccination in Hungary. A principal aim of this research was to determine the concerns, fears, and misunderstandings about COVID-19 vaccinations among individuals who did not comply with vaccination guidelines and declined COVID-19 vaccination. Our findings are intended to guide targeted public health interventions to reduce vaccine hesitancy and increase vaccine uptake.

## Methods

The empirical analysis of this study is based on the data of MASZK study, hosted by the University of Szeged, in which surveys were conducted in Hungary [20]. Data were collected using CATI (Computer-assisted telephone interviewing) methodology between April 2020 and June 2022, once a month, with a sample size of at least 1000 respondents. A multi-step, proportionally stratified, probabilistic sampling procedure was used for sampling, which included both landlines and mobile phone numbers. The sample was representative of the Hungarian population aged 18 years or older by gender, age, education, and type of settlement. Sampling errors were corrected using iterative proportional weighting after the data collection. The data collection was fully complying with the current European and Hungarian privacy data regulations, approved by the Hungarian National Authority for Data Protection and Freedom of Information, and by the Research Ethic Committee of the Medical Research Council of Hungary (resolution number IV/3073-1/2021/EKU). Informed consent was obtained from all survey participants.

This analysis focuses primarily on data collected between February 19, 2021, and June 30, 2022, in 17 surveys (see Table 1), starting from the time when vaccines became widely available. However, the survey also evaluated the willingness to receive vaccination prior to the availability of COVID-19 vaccines to the general public, between December 16-22, 2020. To examine the key trends over time, pandemic waves defined by COVID-19 case numbers have been used [3].

We defined vaccine non-acceptance as an individual decision, at the time of the survey, to decline the COVID-19 vaccine when presented with the opportunity to be vaccinated [21].

To investigate which socio-demographic characteristics are most likely to influence vaccine hesitancy in Hungary, binomial logistic regression models were used. Models were fitted using the glm function of the stats package (version 3.6.2) of R [22]. In order to compare the impact of the socio-demographic predictors across pandemic waves, Average Marginal Effects (AMEs) were calculated [23] using the margins package of R [24, 25].

Multiple-choice questions measured respondents’ self-reported vaccination status and willingness to vaccinate, while reasons for not accepting vaccination were measured with an open-ended survey question. Respondents who reported not having received a single dose of the COVID-19 vaccine were asked the following question, separately for each vaccine type available: “*Do you plan to get vaccinated with the coronavirus vaccine currently available in Hungary from [manufacturer]? (1) yes, as soon as I have the opportunity; (2) yes, but only after some time; (3) no; (4) don’t know.*” In case the mentioned type of vaccine was not yet available in Hungary, the following question was asked: “*If the [manufacturing country]-based [manufacturer] coronavirus vaccine recommended by the health authorities became available, would you be vaccinated with it? (1) yes, as soon as I have the opportunity; (2) yes, but only after some time; (3) no; (4) don’t know.*” If the respondent indicated that they did not plan to receive the listed vaccines, they were asked the following question: “*Please explain in your own words why you do not plan to be vaccinated with any of the vaccines.*”

The responses to the open-ended questions revealed a differentiated picture of the concerns, fears, and misconceptions about COVID-19 vaccines among those who did not accept vaccination. For analysis purposes a category system was developed based on the WHO 5C model, as outlined by Betsch [26], with modifications made to fit the specific context of this study. It was not possible to fully adapt the model, as the questionnaire used only asked about the reasons for vaccine non-acceptance, rather than overall vaccine hesitancy. Thus, we do not have information from those who ultimately decided to take up the vaccine as the question for the reasons of rejection was only asked from those who reported that they have not got the vaccine and neither they plan to take it. Consequently, not all the 5C factors were relevant for this study. Only those factors were selected which could be used in the categorisation of the answers and used these factors in a deductive approach. Nevertheless, there were still answers that could not be classified into these categories. For this reason, an inductive approach was initiated for the classification of these answers. In summary, a mixed approach was employed for the coding of the open-ended responses. Initially, a deductive approach was adopted, with the model subsequently extended through the application of an inductive approach. In the analysis, we therefore developed four pre-defined main categories, including trust barriers, information barriers, risk perception, and other barriers, along with other sub-categories (refer to Table 3 in the Supplementary Material) for identifying reasons for not accepting the COVID-19 vaccination. Table 4 of the Supplementary Material provides an illustrative overview of typical responses categorised into the defined subcategories.

Regarding the reasons for vaccine non-acceptance, 2506 open-ended responses were coded and categorised by two researchers using the pre-defined main and sub-categories. This was carried out by two researchers working independently of each other. During the coding process a response could be coded in more than one category. The pre-defined category system was modified based on the feed-backs from the coding researchers collected after the first 200 responses in the pilot. The degree of agreement between the independent researchers was assessed using Cohen’s Kappa for each category, by accepting at least “moderate” agreement with the Cohen’s Kappa coefficient of >0.4 [27] (refer to Table 3). All disagreements were coded again by a third researcher.

## Results

We categorised data of the 17 surveys into three pandemic waves. Although the time intervals of the data collection do not precisely coincide with the COVID-19 case numbers’ defined pandemic waves [3], this categorization serves as a good approximation and help the interpretation and the understanding of results (Table 1).

**Table 1:** Dates of the data collection and epidemic waves

Pandemic wave	Time of data collection
3^rd^ wave (January 25, 2021 - July 4, 2021)	February 19-25, 2021
	March 24-30, 2021
	April 22-28, 2021
	May 25-31, 2021
	June 22-28, 2021
4^th^ wave (July 5, 2021 - December 26, 2021)	July 23-29, 2021
	August 23-29, 2021
	September 24-30, 2021
	October 24-30, 2021
	November 19-25, 2021
	December 15-21, 2021
5^th^ wave (December 26, 2021 - July 5, 2022)	January 21-27, 2022
	February 23-28, 2022
	March 24-30, 2022
	April 21-24, 2022
	May 25-31, 2022
	June 24-30, 2022

An exceptionally large database with a total sample size of 17,001 was used for the analysis (Table 2). The analysis was complemented by an additional survey data collected on December 16-22, 2020 (n=1000) to compare the examined epidemic waves with the period immediately preceding vaccination. The overall distribution of the sample is representative of the adult population in Hungary (Table 2).

**Table 2:** Proportion of the vaccinated and non-vaccinated respondents in the 3^rd^ to 5^th^ pandemic waves, by socio-demographic characteristics

	3^rd^ wave		4^th^ wave		5^th^ wave		Total
	Vacc.	Non-vacc.	Vacc.	Non-vacc.	Vacc.	Non-vacc.	
*Sex*							
Male	50%	45%	48%	43%	47%	47%	47%
Female	50%	55%	52%	57%	53%	53%	53%
*Age group*							
19-29 yrs	12%	22%	15%	27%	17%	22%	18%
30-39 yrs	13%	20%	16%	21%	16%	23%	17%
40-49 yrs	17%	21%	18%	20%	18%	21%	19%
50-59 yrs	16%	15%	15%	14%	15%	17%	15%
60+ yrs	42%	22%	35%	17%	34%	17%	32%
*Education*							
Elementary	25%	23%	24%	23%	24%	22%	24%
Vocational	23%	20%	20%	26%	21%	26%	22%
High school	30%	37%	32%	38%	32%	38%	33%
Higher education	22%	20%	23%	13%	23%	14%	21%
*Settlement type*							
Capital	20%	16%	20%	9%	20%	8%	18%
County seat	16%	19%	17%	18%	17%	19%	17%
City	34%	37%	34%	39%	35%	38%	35%
Village	30%	29%	29%	34%	29%	34%	30%
*Financial situation*							
Rather poor	32%	33%	28%	36%	29%	36%	30%
Medium	47%	45%	46%	44%	45%	41%	45%
Rather good	21%	22%	26%	20%	26%	23%	25%
**N**	**2366**	**2635**	**4809**	**1191**	**5016**	**984**	**17001**

In December 2020, before vaccines became available for the wider public, only a quarter of the population (24%) expressed willingness to receive the COVID-19 vaccine immediately upon availability. Meanwhile 41% were uncertain, and 35% declined all forms of COVID-19 vaccinations.

**Fig. 1:**
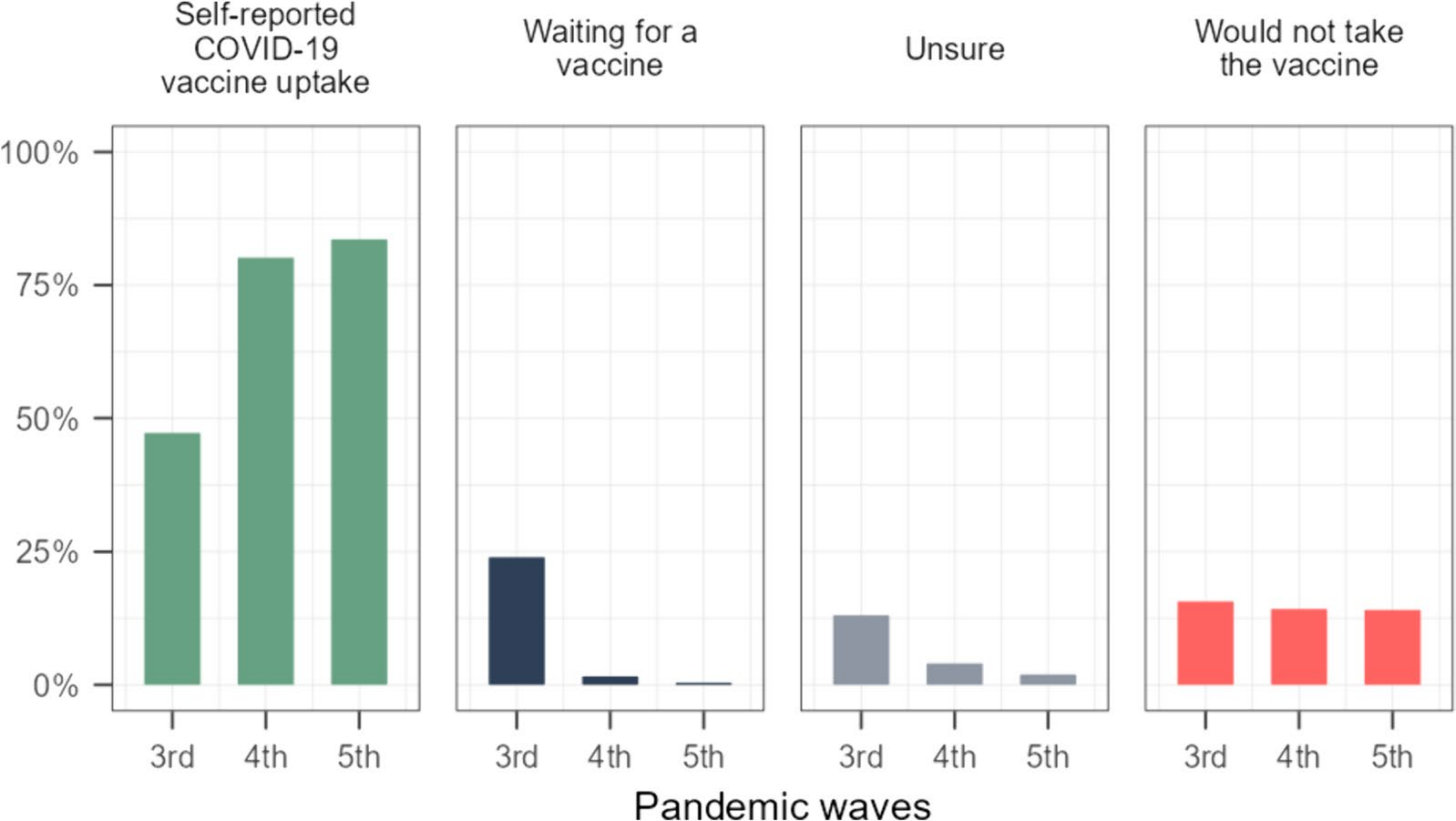
Self-reported COVID-19 vaccination attitudes in Hungary during the 3^rd^ - 5^th^ pandemic waves based on the results of monthly representative cross-sectional surveys

During the third wave of the pandemic (25^th^ January 2021 - 4^th^ July 2021), following the commencement of the COVID-19 vaccination campaign, 47% of people self-reported that they had received a single dose of vaccine and 16% would not be taking any type of vaccine. A significant proportion of individuals were either unsure whether they would be vaccinated (13%) or were waiting to be vaccinated (24%) (Fig. 1). By the fourth wave of the pandemic in 2021, the percentage of individuals waiting for vaccination and those uncertain about receiving the vaccine had almost disappeared (2 and 4%, respectively), and the majority of Hungarian society was basically divided into two groups, those who reported receiving the single-dose vaccine (80%) and those who declined the vaccine (14%). The percentage of individuals who reported not accepting the vaccine remained almost unchanged during the three waves of the pandemic, 16% in the third wave and 14-14% in the fourth and fifth waves of the pandemic (Fig. 1).

**Fig. 2:**
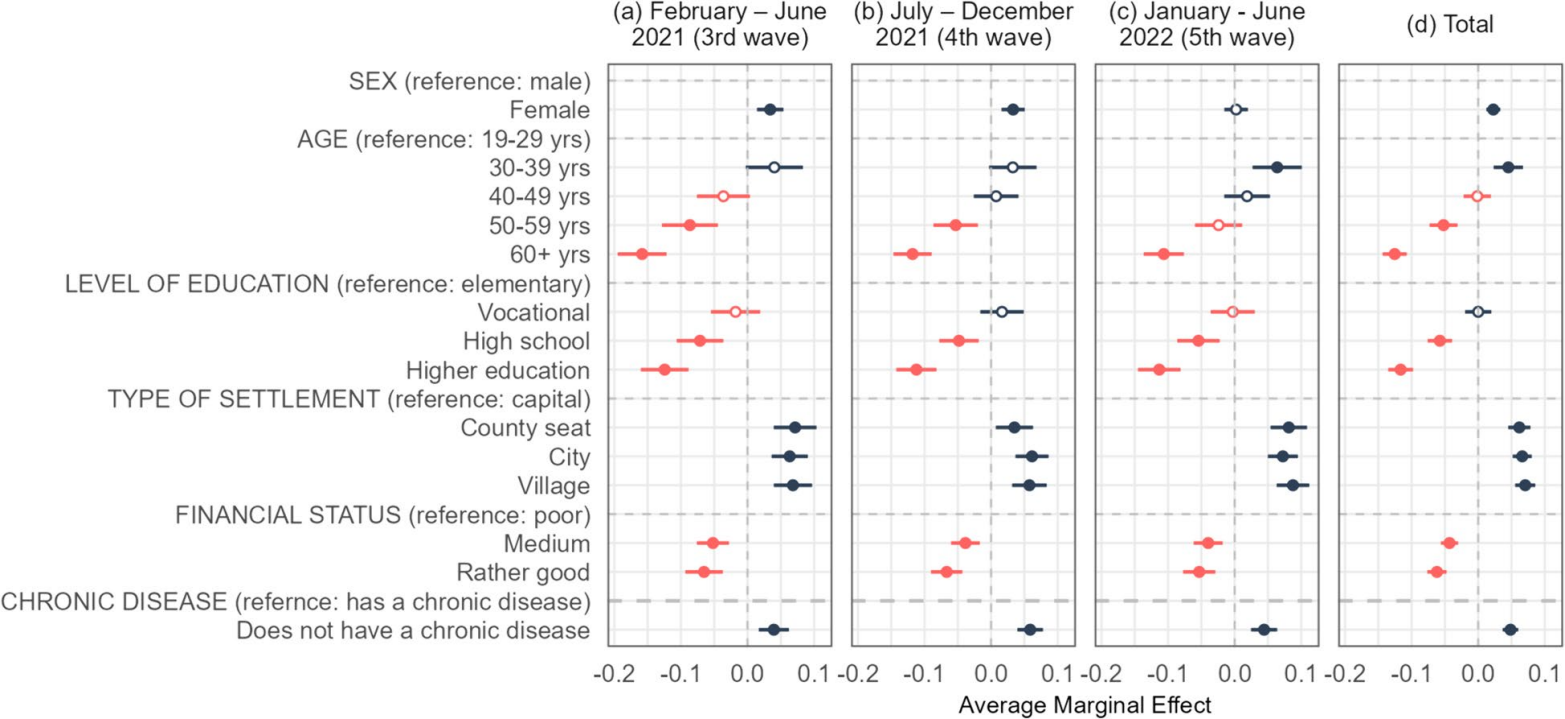
The socio-demographic determinants of vaccine non-acceptance in Hungary during the 3^rd^ to 5^th^ pandemic COVID-19 waves (binomial logistic regressions, average marginal effects)

Socio-demographic characteristics were important determinants of the observed vaccine hesitancy. In the 3^rd^ and 4^th^ pandemic waves (and overall, considering all the waves together), women were more likely to be vaccine-hesitant than men, although this significant difference disappeared in the 5^th^ wave. For all waves, individuals in younger age groups (especially the 30-39 years age category) and those with lower levels of education and lower income were more likely to decline vaccination, while those with chronic disease and those living in the capital city were the least likely. The social factors behind vaccine non-acceptance were relatively stable over time with only minor changes following the introduction of COVID-19 vaccines (Fig. 2).

**Fig. 3:**
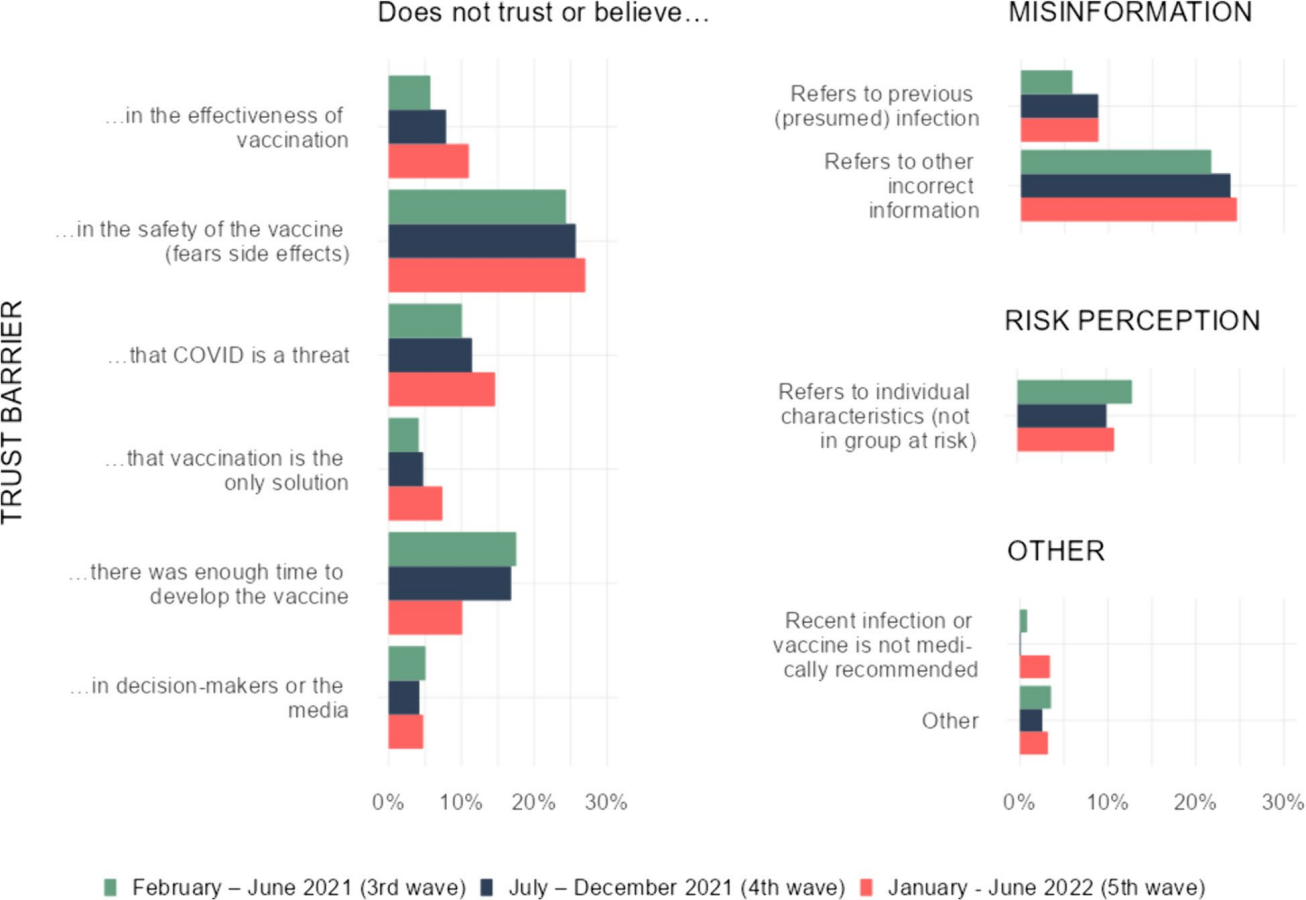
The reasons for vaccine hesitancy among vaccine non-accepters in Hungary during the 3^rd^ to 5^th^ pandemic COVID-19 waves

Of the 2481 open responses, 2270 responses were categorised. The Cohen’s Kappa values ranged from 0.41 to 0.92 (for concrete values, see Table 3). Out of the main categories (trust barrier, information barrier, risk perception and other barriers), a significant reason behind vaccine refusal can undoubtedly be identified as lack of trust, specifically distrust in science. 68% of the participants identified trust barrier as one of the reasons for vaccine hesitancy. Among vaccine non-accepters a significant majority (66%) attributed their hesitancy to the lack of trust in science. This compares with 39% of respondents who also mentioned facing an information barrier, whereas only 5% associated with any other structural or individual reason (Fig. 3).

Although overall the trust barrier remains stable over time, its composition varies significantly across the three pandemic waves analysed. The reasoning that “the vaccine was developed too quickly” became less popular among responders, while confidence in the effectiveness and safety of the vaccine were declining (although changes in safety were not significant), with fewer people perceiving COVID-19 as a threat. It can also be seen that, as time progresses an increasing number of people believe that COVID-19 vaccination is the sole means of protection. Overall, the categories related to trust in science (and of all the categories surveyed), fear of side effects was the most common reason for not accepting vaccination, mentioned by 26% of the respondents.

Political views are not highlighted in the results, as this study did not focus on them. However, the proportion of respondents was around 4-5% in each wave who did not accept vaccination mentioned the lack of trust in decision-makers or media as a reason for their decision.

Incorrect information was the second most common answer. This category comprised of responses indicating that the decision was based on misinformation. The most prevalent sub-category was where the respondents referred to self-declared past infections (albeit not recent). The proportion of reference to previous infection significantly increased in the 4^th^ and 5^th^ waves (6% of respondents mentioned previous infection during the 3^rd^ wave, compared to 9-9% during the 4^th^ and 5^th^ waves). Additionally, the proportion of responses that fell outside of this subcategory but were identified as a type of information barrier increased in subsequent waves.

The third category was risk perception, where the respondents considered themselves not to be at risk from the virus on account of their own attributes (e.g. youth, robust immune system). The reference to this answer decreased over time (although not significantly).

## Discussion

This large-scale, representative study used monthly cross-sectional surveys to show changes in public attitudes towards COVID-19 vaccination during the major pandemic waves in Hungary.

There was a substantial shift in attitudes towards COVID-19 vaccination from December 2020 to December 2021 in Hungary. In December 2020 - before the study period and prior to the introduction of the vaccines, - vaccine hesitancy was at a high level in Hungary with a refusal rate of 35% and 41% of the population expressing uncertainty. However, after the introduction of the vaccines, there was a significant decrease in the ratio of vaccine hesitant groups (who were unsure or wanted to wait with vaccination) over time. At the same time, the ratio of vaccine non-acceptors (in our measure, those who have neither got, nor been hesitant to take the vaccine, but completely reject it) remained almost constant over the study period. During the third pandemic wave, vaccine non-acceptance was 16%, while it was 14-14% during the fourth and fifth waves. Our data (and administrative data as well - see Figure 4 in the Supplementary Material) indicates that a plateau was reached in the number of individuals who were willing to be vaccinated by the end of 2021, despite the availability of COVID-19 vaccines in Hungary. This finding remains crucial as the situation has not changed since then, and COVID-19 vaccination coverage with primary series doses has not increased in the following years in Hungary [2].

Hungary has a well-established history of successful disease prevention through compulsory vaccination programs, and thus childhood vaccination coverage is particularly high [28]. However, a high uptake of compulsory childhood vaccinations does not necessarily mean that positive attitudes towards vaccination extend to voluntary vaccinations. Voluntary adult vaccines, such as the influenza and COVID-19 vaccines, are less well accepted in Hungary [29, 30].

Our study showed, that the acceptance of the COVID-19 vaccine exceeded that typically observed for voluntary adult vaccinations such as the influenza in Hungary, probably due to initial widespread vaccination communication and vaccine-related benefits (e.g. COVID passport). Nonetheless, the vaccination coverage was increasingly lagging behind the EU average, and it was less able to markedly reduce virus transmission and protect vulnerable groups during pandemic surges. Since the primary series vaccination campaign, willingness to receive a COVID-19 booster vaccination has drastically decreased in Hungary [2]. A limitation of this study, that it only analyses the first dose of vaccination. For individuals aged 18 years and older, the difference in vaccination coverage between the first and second doses of COVID-19 is only 2.6 percentage point, so our results are likely to apply well to the group of those not receiving the whole primary vaccination series in Hungary, but not for those not accepting the booster doses. Self-reported vaccine acceptance or willingness to receive COVID-19 vaccination may not be a reliable predictor of real-world vaccine uptake, as noted in the study by Andrejko et al. [31]. In June 2022, 26% of Hungarian adults had not received a COVID-19 vaccine dose [2]. Nonetheless, our findings indicate that the proportion of unvaccinated individuals during that time was only 18% according to self-reporting. It is possible that the overestimation of the percentage of vaccinated individuals in our study was due to selection bias. Individuals who are more likely to be concerned about the pandemic and therefore more likely to be vaccineted are also more likely to participate in COVID-19-related surveys. Furthermore, social desirability bias may lead some unvaccinated individuals to claim they are vaccinated [4, 32]. Due to this biases, despite striving to produce nationally representative outcomes through the design of the sampling methods and data weighting, the respondents may not fully represent the general adult population in Hungary. However, these biasing mechanisms were assumed to remain constant over time, making the data suitable for trend analysis.

Our results indicate that reluctance to receive COVID-19 vaccination was most prevalent among younger adults, and those with lower educational attainment or financial status, those with chronic disease, and those residing outside of the capital. These results are in line with the existing literature [33–37]. A previous ecological study has already suggested a similar association in Hungary [3]. Thus, socioeconomic inequalities strongly influence vaccination attitudes in Hungary, and this association appears to be stable over time. The difference in COVID-19 vaccination coverage between Hungary and the EU may be partly explained by the higher proportion of the socio-economically deprived population in Hungary.

The study found that the main reasons for vaccine hesitancy were a lack of trust, particularly in science, and information barriers. In East-Central Europe, institutional trust, including trust in health institutions, has historically been low [38], which was further reduced by the COVID-19 pandemic [39]. The findings are consistent with other studies that highlight the link between a lack of trust (both in general and specifically in authorities, institutions, and science), and vaccine hesitant attitudes and behaviours [18, 19]. In interpreting the research results, it is important to note that the WHO vaccination recommendation at the time of the study was used to ensure that primary vaccination was recommended in the first line for those at the highest risk of severe COVID-19, then, if vaccines were plentifully available, as was the case in Hungary, for the high-risk group, followed by the medium-risk group (which contains all healthy adults) [40]. Furthermore, the WHO recommended vaccination regardless of previous infection [40, 41], with a specified time interval between the vaccination and the previous infection. These guidelines are especially important in the interpretation of the misinformation category of the reason for non vaccination, especially in the subcategory ’refers to previous (presumed) infection’. Although we could move out some of those respondents from the misinformation category, who have been infected in the last four months (and move them to the fifth, other category), but as the questionnaire only asked for the first PCR test, we could not detect those who has been infected twice, or who detected the infection with other types of tests. Therefore, in this study it was considered misinformed for previously infected individuals to decline vaccination against COVID-19 partially on the basis of naturally acquired immunity.

The results show that while the overall trust barrier remains stable over time, its composition varies significantly across the three pandemic waves analysed. The third wave of the pandemic was the most severe in Hungary in terms of the number of severe illnesses and deaths recorded, moreover, by this time pandemic fatigue had set in. Non-pharmacological measures were gradually withdrawn with the introduction of vaccination [42]. Towards the end of the research period, the Omicron variant emerged, causing a milder course of disease than the previously dominant Delta variant [43]. The reason for vaccine refusal was increasingly the fact that respondents no longer felt threatened by COVID-19. As public experience with vaccination increased, the argument that the vaccine had been developed too quickly became less prevalent among vaccine hesitants, while more people believed that the vaccine was ineffective as it became clear to people that they could still become infected and even transmit the virus despite being vaccinated [44, 45]. As the pandemic progressed, more and more people mistakenly believed that the risks of vaccination outweighed the benefits, and concerns about vaccine safety became the most frequently cited argument, with its share rising steadily.

## Conclusion

In this paper we analysed large-scale, representative monthly cross-sectional survey data to reveal changes in public attitudes towards COVID-19 vaccination from the beginning of the vaccination campaign in Hungary. Vaccine hesitancy related to COVID-19 vaccination heavily decreased from December 2020 to December 2021 in Hungary, however the size of the group who radically rejected vaccination did not change over time. Socio-demographic characteristics were an important determinant of the observed vaccine hesitancy in each observed pandemic waves. The analysis of the reasons for vaccine rejection showed that the main reasons behind not acceptance are the lack of trust (especially distrust in science) and having misinformation. Identifying and understanding the complexity of how vaccine hesitancy evolved during the pandemic can help to understand and halt the decline in both COVID-19 and general vaccine confidence by developing targeted public health programs to address these issues.

### Supplementary Information

Supplementary Material 1.

## Data Availability

The datasets used in the current study are available from the corresponding author upon reasonable request.
