# Supplementary material for: Socioeconomic determinants and reasons for non-acceptance to vaccination recommendations during the 3rd - 5th waves of the COVID-19 pandemic in Hungary
Source: BMC Public Health. 2024 Jul 5;24:1796. doi: 10.1186/s12889-024-19267-2 (PMC11225232; doi:10.1186/s12889-024-19267-2)
Supplement: Supplementary file 1 — Supplementary Material 1. [file 12889_2024_19267_MOESM1_ESM.docx]

Figure 4. The cumulative ratio of those who got the (a) primary course and (b) the first booster of any COVID-19 vaccines in the adult population of Hungary between 2020 December and 2023 September (source: ECDC Vaccine Tracker)


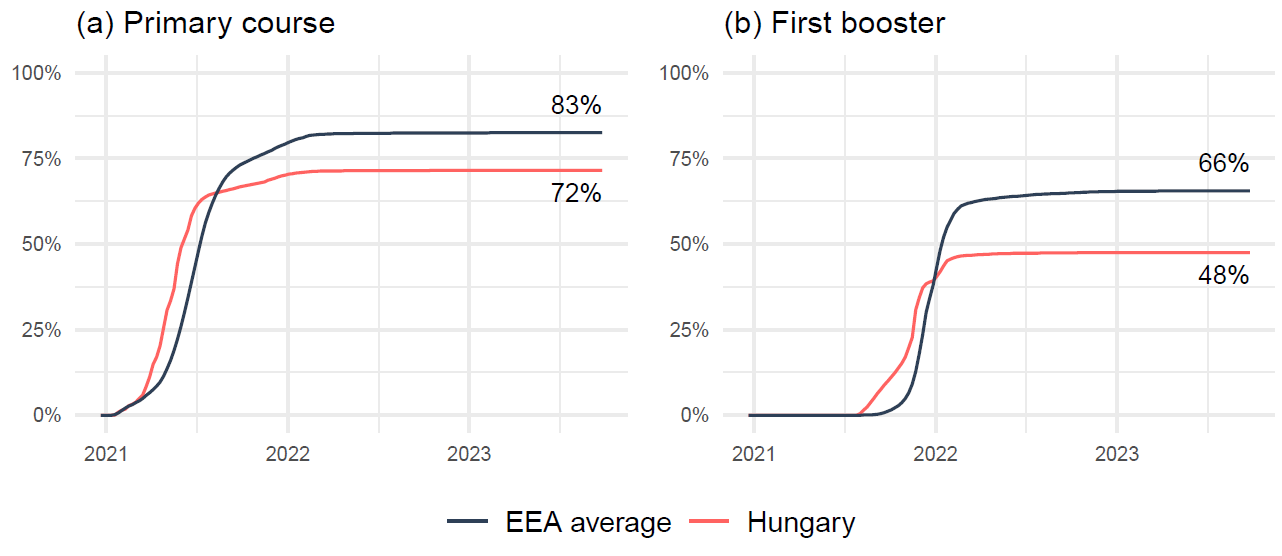


Table 3. Cohen’s Kappa values of the categorised open-ended questions measuring the reasons for vaccine hesitancy among vaccine non-accepters

|  | Agreement rate | Cohen’s Kappa |
| --- | --- | --- |
| 1. Trust barrier |  |  |
| 1.1. Does not trust/believes in science |  |  |
| 1.1.1. Does not trust/believe in the effectiveness of vaccination | 96.6% | 0.76 |
| 1.1.2. Does not trust/believes in the safety of the vaccine, fears side effects | 91.8% | 0.78 |
| 1.1.3. Does not believe that COVID is a threat | 91.3% | 0.58 |
| 1.1.4. Does not believe that vaccination is the only solution | 98.1% | 0.83 |
| 1.1.5. The vaccine was developed too quickly | 96.6% | 0.87 |
| 1.1.6. Overall distrust in science | 87.7% | 0.52 |
| 1.2. Does not trust/believe in decision makers or media |  |  |
| 1.2.1. Decision makers | 95.8% | 0.41 |
| 1.2.2 . Media | 98.6% | 0.44 |
| 2. Information barrier | 83.5% | 0.56 |
| 2.1. Refusing vaccination due to previous (presumed) infection | 98.7% | 0.92 |
| 3. Risk perception |  |  |
| 3.1. Does not belong to group at risk due to a specific characteristic (e.g. young, have a good immune system) | 95.0% | 0.74 |
| 4. Other individual reasons (e.g. accessibility, fear of needles, vaccine is not compulsory) |  |  |
| 4.1. Accessibility | 99.6% | 0.47 |
| 4.2. Fear of needles, fear of pain | 98.7% | 0.58 |
| 4.3. Vaccine is not compulsory | 99.5% | 0.87 |
| 5. Other | 98.0% | 0.67 |

Table 4. Some examples of open-ended responses for each category of analysis

| 1.1.1. Does not trust/believe in the effectiveness of vaccination | “It doesn’t protect against coronavirus because everyone who has been vaccinated is sick.”  “No medical research shows that the vaccine protects against the virus.”  “Some have been vaccinated and have already had the virus twice.” |
| --- | --- |
| 1.1.2. Does not trust/believes in the safety of the vaccine, fears side effects | “I've heard of a lot of side effects. People I know also complain about the side effects.”  “The long-term consequences have not been assessed.”  “I don’t know the composition of vaccines and the side effects in the long term.” |
| 1.1.3. Does not believe that COVID is a threat | “This is just being blown up.”  “Not as dangerous as they say, it's just another flu.”  “The virus isn't strong enough, it's bearable. It's all just exaggerated.” |
| 1.1.4. Does not believe that vaccination is the only solution | “I believe in strengthening the immune system.”  “I try to avoid it in other ways, like avoiding the crowds.”  “I'd rather live a healthy life and try to avoid getting sick in the first place. I agree with the social distancing and mask wearing advice.” |
| 1.1.5. The vaccine was developed too quickly | “I am not a guinea pig.”  “This vaccination was not sufficiently tested.”  “I hear a lot of rumours about it, I don't trust it because it's in the experimental phase.” |
| 1.1.6. Overall distrust in science | “I don't trust vaccines.”  “I am against all medicines”.  “I do not trust them because of their weakened pathogen content.” |
| 1.2.1. Does not trust/believes in decision makers | “I sense a lot of politics in it”  “I don't trust it because they are very aggressive in wanting people to vaccinate themselves.”  “Nobody takes responsibility for the possible side effects of the vaccine.” |
| 1.2.2. Does not trust/believes in media | “There was a lot of propaganda.”  “On Facebook you can read one piece of information, on TV you can hear another, on each of them they say different things. If everyone said the same thing, I might think about vaccination.”  “Because they lie” |
| 2.1. Refusing vaccination due to previous (presumed) infection | “I'm pretty sure I've already had Covid”  “I've had symptoms of the virus before, I think I've been through it, so I've got some immunity in my system”  “I've been through the coronavirus, so it's no longer a threat to me.” |
| 3.1. Does not belong to group at risk due to a specific characteristic (e.g. young, have a good immune system) | “I am not in a risk group; I am very young.”  “I eat healthily, exercise, take vitamins. I trust myself and my immune system more than I trust vaccines.”  “I don't have any diseases, there's not much chance of me getting infected.” |
| 4.1. Accessibility | “I applied for it, but the doctor spoke to me in a condescending way.”  “I wouldn't be in line yet anyway.”  “No one to register me.” |
| 4.2. Fear of needles, fear of pain | “I have a phobia of needles”  “I don’t like needles.” |
| 4.3. Vaccine is not compulsory | “I will vaccinate myself when it becomes compulsory”  “I will wait until the vaccination is demanded.” |
